# Ovine paratuberculosis: a confirmed case of Johne’s disease in Libya

**Published:** 2013-11-20

**Authors:** M.A.M. Sharif, M.E. Farhat, E.S. Kraim, N.A. Altrabulsi, A.M. Kammon, A.S. Dayhum, I.M. Eldaghayes

**Affiliations:** 1 *Department of Pathology, Faculty of Veterinary Medicine, University of Omar Al-Mukhtar, Albeida, Libya*; 2 *National Center of Animal Health (NCAH), Zawia, Libya*; 3 *Faculty of Veterinary Medicine, University of Tripoli, P.O.Box 13662, Tripoli, Libya*

**Keywords:** Johne’s disease, Libya, Mycobacterium, Ovine, Paratuberculosis

## Abstract

Paratuberculosis (Johne’s disease) was suspected in a herd of approximately 300 sheep after weight loss and scouring had increased in adult animals despite repeated treatment with anthelmintics, antibiotics, multivitamins and minerals. The herd is located near Tarhouna city. Herd history revealed that a total of 60 ewes showed clinical symptoms and deaths during the last two years. The last case that we attended was submitted to the National Center of Animal Health (NCAH) for a detailed laboratory examination. Gross pathological and histological examination of tissue samples revealed results that were highly comparable with Johne’s disease. A definitive diagnosis was made only by histopathological identification of *Mycobacterium paratuberculosis* in the intestines using Ziehl-Neelsen stain. This is the first documented case of *M. paratuberculosis* in sheep in Libya.

## Introduction

Ruminant Paratuberculosis (Johne’s disease) (JD) is a chronic infectious enteric disease that is characterized by persistent diarrhea, progressive debilitation and poor response to therapy. The disease has a worldwide prevalence and it has been reported to affect domestic, wild and zoo ruminants (Buergelt and Ginn, 2000; Hope *et al.*, 2000; Kruze *et al.*, 2006; Salem *et al.*, 2013). The etiological agent is an acid-fast bacillus known as *Mycobacterium avium* subsp. *paratuberculosis* that has also been suspected to cause regional ileitis or Crohn’s disease in humans (Erume *et al.*, 2001; Kaevska and Hruska, 2010; Singh and Gopinath, 2011).

Paratuberculosis in animals exists in two distinct forms; the lepromatous or multibacillary form in which the macrophages are stuffed with bacilli (Biplab *et al.*, 2010) and the paucibacillary form in which lymphocytes are predominantly present with or without bacilli in intestinal mucosa (Clark *et al.*, 2010). The affected animals shed large quantities of the microorganism in their milk and feces thus contaminating the pasture and farm premises (Hulten *et al.*, 2001). Unfortunately, the disease is still incurable due to lack of proper therapies (Jones, 1989; Maxie *et al.*, 2007; Singh *et al.*, 2011).

A wide array of procedures and laboratory tests ranging from conventional methods like skin sensitivity test, Ziehl-Neelsen stained smears, and histopathological analysis of terminal segment of the ileum and mesenteric lymph nodes (Buergelt and Ginn, 2000) have been employed for diagnosis. Furthermore, histological examination is also a reliable indicator for the disease (Kurade *et al.*, 2004; Sikandar *et al.*, 2012).

## Case Details

In June 2013, a sheep herd consisting of approximately 300 heads, located in Daoon Vilage, near Tarhona city in Libya was visited upon the request from the owner who noticed a prolonged problem. The owner complaint was as follows: loss of 60 adult animals due to severe emaciation, bottle jaw followed by diarrhoea and death during the last two years. The animals did not respond to the repeated treatment with anthelmintics, antibiotics, multivitamins and minerals.

To definitively diagnose the disease, one of the ailing animals was slaughtered and the post-mortem examination was performed in the National Center of Animal Health (NCAH) laboratory in Tripoli.

The excessive mesenteric attachments were trimmed off both from the intestines and lymph nodes. Later on, the samples were cut into suitable segments. Fixing was carried out using 10% neutral buffered formalin. Samples were subjected to further processing steps that included dehydration, clearing, embedding, sectioning to 0.5 μm thickness and routine Haematoxylin & Eosin staining (Luna, 1968). Slides were also stained by Acid-fast stain (Ziehl-Neelsen stain). All slides were carefully examined using light microscopy.

### Gross pathology

The animal showed classical clinical signs of ovine paratuberculosis that include: severe emaciation, dry and rough woolly coat, and with a clear evidence of diarrhea.

**Fig. 1:**
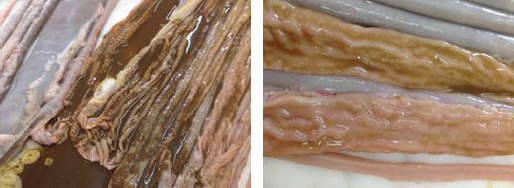
Left: Morphological appearance of paratuberculosis lesions in the ileum and jejunum of sheep clearly show thickening and corrugations of the mucosa. Right: A closer view of the lesions.

The most striking gross lesions observed were the mucosal corrugations of the ileum and jejunum (Fig. 1) as well as chronic lymphadenitis of the mesenteric lymph nodes.

In addition, two Tapeworms (Moniezia) were detected in the small intestine along with one small focal hemorrhagic lesion in mucosal wall of the cecum.

### Histopathology

**Fig. 2:**
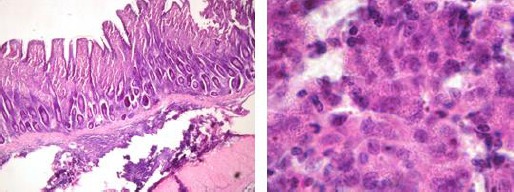
Left: Small Intestine, epithelioid macrophages with foamy cytoplasm and eccentric nuclei are seen in the mucosa. H & E (100X). Right: Higher magnification (1000X).

The intestinal walls of the ileum and jejunum were extremely thickened. Extensive infiltration by mononuclear cells (epithelioid-macrophages, plasma cells and lymphocytes) were observed especially in the mucosa and upper portions of submucosa of intestinal sections. Cytoplasm of the epithelioid macrophages was foamy in appearance and darker round to oval shaped nuclei were eccentric in their position (Fig. 2).

Few eosinophils and neutrophils had also infiltrated. Crypts of Lieberkuhn (intestinal mucosal glands) were atrophied. Lumen of some of the crypts was filled with exfoliated cells mixed with inflammatory cells. Infiltration by macrophages was also observed in the wall of crypts. The lamina propria of the mucosa was abundantly puffed-up with mononuclear cells. A mild mononuclear cell infiltration was also observed in serosa.

**Fig. 3:**
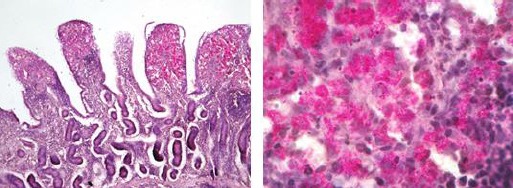
Left: The mucosa of ileum of small intestine is infiltrated with epithelioid macrophages that are stained rose-red to pink AFB. ZN (200X). Right: Higher magnification (1000X).

Using the Acid-fast stain, we were able to detect that the cytoplasm of epithelioid macrophages was heavily stuffed with microorganisms. The appearance of microorganisms in macrophages was red to pink coloured. Laden macrophages were mostly present in the mucosa; however, small aggregates also existed in the upper submucosa (Fig. 3).

Mesenteric lymph nodes had thick capsules of fibrous connective tissue. Epithelioid macrophages also occupied most of the cortical areas of mesenteric lymph nodes. Micro-granulomas were present in parenchyma of lymph nodes as well. Fibrous connective tissue followed by a zone of mononuclear cells was noticeable around the granulomas.

## Discussion

The present study revealed definitive evidences of classical intestinal lesions that are characteristic of JD in sheep. Our results are in accordance with those of (Maxie *et al.*, 2007; Alharbi *et al.*, 2012) who observed that advanced cases of JD were typified by diffused intestinal thickening coupled with longitudinal and transverse corrugations that gave rise to asymmetrical folds having red coloured surfaces, but no ulcerations. In our case, caseous necrosis and calcification of mesenteric lymph nodes were not found, an observation that is contrary to the findings documented earlier (Kheirandish *et al.*, 2009; Sikandar *et al.*, 2012).

Previously, histopathological examination has been reported as a better indicator for the diagnosis of ovine paratuberculosis (Kurade *et al.*, 2004; Hailat *et al.*, 2010) and our current study also concurs with this verity. Mucosal thickening and corrugations occurring due to mononuclear cell infiltration and edema in the mucosa and submucosa (Maxie *et al.*, 2007) were observed in our case (Fig. 1). Our results are in conformity with the findings of former studies that reported the occurrence of epithelioid cells along with MNCs (Al-Dubaib and Mahmoud, 2008).

Hypotheses and evidence about the relationship of this pathogen in animals and humans must be taken into account (Chamberlin *et al.*, 2001; Naser *et al.*, 2004; Abendaño *et al.*, 2013; Rhodes *et al.*, 2013). Discrete infection among human population indicates that this bacterium has the potential to contaminate minced meat intended for human consumption and thus act as a source for human exposure to *M. avium* subspecies *paratuberculosis* (Collins, 2003; Antognoli *et al.*, 2008).

Therefore, appropriate hygienic measures are critical to safeguard human population against meat-borne *M. paratuberculosis* infections. Unfortunately, the existing measures are insufficient to completely avert the infected tissues from contaminating the human food (Antognoli *et al.*, 2008).

Despite the two mentioned reports about paratuberculosis, according to our research in the literature (Muhammed and Ivoghli, 1983; Mustafa and Mugadmi, 1986), where the first was an experimental study and the second was about the disease in cattle, the case we present here is considered to be the first reported case of ovine JD in Libya.

Therefore, we strongly believe that provision of adequate information concerning the epidemiological and pathological trends of JD in domestic animals in Libya will facilitate the consideration of this disease on a top priority basis and will significantly contribute towards the acquisition of optimal control measures including vaccination programs that are usually followed in some countries (Reddacliff *et al.*, 2006).

## Conclusions

Due to the absence of the database of the infectious diseases in Libya, and no previous reports about this disease, we are raising the alarm to alert against this life-threatening disease and suggest that there is an immediate need to develop an effective strategic plan at the national level to combat paratuberculosis in small ruminants.

Many methods such as ELISA and PCR tests were used to confirm the disease in animals (Botsaris *et al.*, 2013; Pruvot *et al.*, 2013).

However, the importance of our present study is that it has accentuated the significance of a careful histopathological appraisal using intestinal samples and mesenteric lymph nodes for the diagnosis of paratuberculosis in sheep.

Despite being relatively a time-consuming process, the histopathological examination is preferred over the other conventional and serological protocols by virtue of its low cost and a higher specificity. It is considered as an efficient diagnostic tool that can be routinely performed for the diagnosis of JD (Kurade *et al.*, 2004; Hailat *et al.*, 2010).
